# PAG/Cbp suppression reveals a contribution of CTLA-4 to setting the activation threshold in T cells

**DOI:** 10.1186/1478-811X-11-28

**Published:** 2013-04-19

**Authors:** Michal Smida, Clemens Cammann, Slavyana Gurbiel, Nadja Kerstin, Holger Lingel, Sabine Lindquist, Luca Simeoni, Monika C Brunner-Weinzierl, Miloslav Suchanek, Burkhart Schraven, Jonathan A Lindquist

**Affiliations:** 1 Institute of Molecular and Clinical Immunology, Otto-von-Guericke University, Leipziger Strasse 44, Magdeburg, 39120, Germany; 2 Current address: Center for Molecular Medicine, Lazarettgasse 14, AKH BT 25.3, Vienna, 1090, Austria; 3 Department of Experimental Pediatrics, Otto-von-Guericke University, Leipziger Strasse 44, Magdeburg, 39120, Germany; 4 Department of Neurology, Hannover Medical School, Carl-Neuberg-Str.1, Hannover, 30625, Germany; 5 Department of Neurochemistry and Molecular Biology, Leibniz-Institute for Neurobiology, Brenneckestr. 6, Magdeburg, 39118, Germany; 6 EXBIO Praha, a.s., Nad Safinou II 341, Vestec, 252 42, Czech Republic; 7 Department of Immune Control, Helmholtz Centre for Infection Research, Braunschweig, Germany; 8 Current address: Department of Nephrology and Hypertension, Diabetes and Endocrinology, Otto-von-Guericke University Magdeburg, Leipziger Strasse 44, Magdeburg, 39120, Germany

**Keywords:** Human, T cells, Protein kinases, Cell activation, Tolerance

## Abstract

**Background:**

PAG/Cbp represents a ubiquitous mechanism for regulating Src family kinases by recruiting Csk to the plasma membrane, thereby controlling cellular activation. Since Src kinases are known oncogenes, we used RNA interference in primary human T cells to test whether the loss of PAG resulted in lymphocyte transformation.

**Results:**

PAG-depletion enhanced Src kinase activity and augmented proximal T-cell receptor signaling; exactly the phenotype expected for loss of this negative regulator. Surprisingly, rather than becoming hyper-proliferative, PAG-suppressed T cells became unresponsive. This was mediated by a Fyn-dependent hyper-phosphorylation of the inhibitory receptor CTLA-4, which recruited the protein tyrosine phosphatase Shp-1 to lipid rafts. Co-suppression of CTLA-4 abrogates this inhibition and restores proliferation to T cells.

**Conclusion:**

We have identified a fail-safe mechanism as well as a novel contribution of CTLA-4 to setting the activation threshold in T cells.

## Background

T cells orchestrate the adaptive immune response. However, to fulfill this function they must first be activated by specific antigenic peptides presented by MHC molecules. How T cells are able to distinguish self antigens from foreign is an important question, as inappropriate activation may lead to autoimmunity. It was long held that self-reactive T cells are deleted in the thymus. However, the presence of mechanisms that induce peripheral tolerance, as well as the observation that both peripheral conventional and regulatory T cells can be self-reactive has challenged this view. Although it is now accepted that autoreactive T cells escape the thymus [1], it remains unclear how the threshold for activation is set to ensure that these potentially destructive cells remain quiescent. The activation threshold for T cells appears to be determined by a number of TCR-induced proximal feedback loops [2]. One such loop involves the tight control over the activation of Src family kinases, Lck and Fyn [3], by the phosphoprotein associated with glycosphingolipid-enriched microdomains (PAG), also known as the Csk binding protein (Cbp); a transmembrane adaptor protein [hereafter referred to as PAG].

The primary function of PAG appears to be recruiting the C-terminal Src kinase (Csk) to the plasma membrane, where Csk then phosphorylates the inhibitory tyrosines located near to the carboxy terminus of the Src kinases [4-6]. The phosphorylated C-terminus binds intramolecularly to the SH2 domain leading to the formation of a closed-inactive conformation [7-9]. In agreement with this mechanism, PAG over-expression inhibits T-cell activation [4,10].

As PAG, Csk, and the Src kinases are all ubiquitously expressed proteins, this regulatory circuit may be important in many cellular systems. Indeed, PAG has been demonstrated to regulate cellular transformation. In *Theileria*-transformed B cells, the loss of PAG was shown to positively correlate with proliferation [11]. Whereas in B-cell Non-Hodgkin’s Lymphoma (B-NHL) PAG contributes to transformation by maintaining Lyn in an open conformation [12]. Recently, PAG was also described as a tumor suppressor, due to its ability to bind and thereby sequester Src kinases away from their substrates [13]. However, the exact role of PAG in regulating transformation appears to be context-dependent, as PAG has been demonstrated to be absent or present in a number of lymphomas [14].

In addition to inhibiting Src kinases, we have also shown that PAG negatively regulates Ras [15]. PAG also restricts the mobility of lipid rafts within the membrane via its binding to the cytoskeletal adaptor EBP-50 [16,17]. Since the data suggest that PAG is an important negative regulator, a *Pag1* knockout was predicted to have a severe phenotype, as the *Csk* knockout is embryonic lethal [18,19]. However, as *Pag1* knockout mice possess no apparent defects, the importance of PAG as a negative regulator has been questioned [20,21]. Our investigation of these mice revealed that a constitutive *Pag1* knockout develops a compensatory mechanism [22], suggesting that the use of conventional knockout mice is not the best strategy to investigate PAG function. Therefore to address whether the loss of PAG would result in lymphocyte transformation, we used RNA inhibition to investigate PAG function in primary human T cells.

As the suppression of murine PAG expression by siRNA was previously reported in fibroblasts [23], we utilized the corresponding RNA-sequences to target human PAG [15,24]. We found that PAG suppression in human T cells led to enhanced Src kinase activity, which was reflected by increased phosphorylation of the activatory tyrosine. Additionally, we detected both enhanced basal tyrosine phosphorylation, as well as an enhanced TCR-induced phosphorylation, including the activation of key proteins such as ZAP-70 and PLCγ1. However despite showing enhanced proximal signaling, the proliferation of PAG-deficient cells was dramatically reduced. Thus, it appears that other negative regulatory feedback loops have been activated that induced a state of unresponsiveness within these T cells. We further show that this involves a negative feedback loop via the inhibitory receptor CTLA-4, which recruits the phosphatase Shp1, and, in this way, prevents strong proximal signals from being translated into enhanced T-cell activation.

## Results

### PAG suppression enhances proximal signaling in human T cells

**Figure 1:**
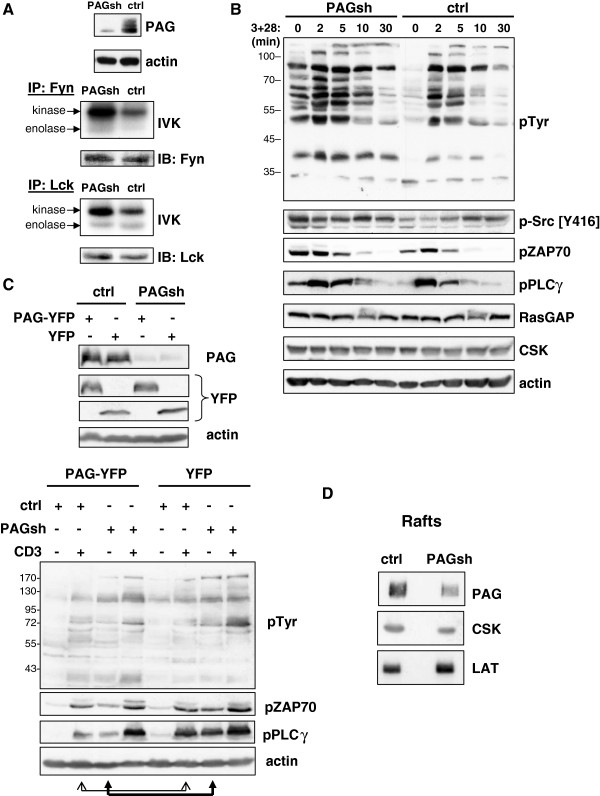
**PAG down-regulation enhances Src kinase activity and proximal signaling.** (**A**) Jurkat T cells were transfected with either non-targeting shRNA (ctrl) or PAGshRNA (PAGsh) and three days later lysed. The top panel shows the PAG downregulation. The Src family kinases Fyn (middle) and Lck (bottom) were immunoprecipitated and *in vitro* kinase assay (IVK) performed. Phosphorylation was visualized by autoradiography, the amount of precipitated kinase was determined by immunoblotting. Quantification of the kinase bands can be found in Additional file 2: Figure S2. (**B**) The cells from (**A**) were stimulated with CD3 and CD28 antibodies as indicated and lysates immunoblotted with specific antibodies. Actin staining is shown as a loading control. Quantification can be found in the supplemental figure. (**C**) Jurkat T cells were transfected with either control or PAG shRNA and co-transfected with either PAG-YFP, which differs by two nucleotides within the targeting region, or YFP alone. The YFP-tag was included to allow us to distinguish between the over-expressed protein and endogenous PAG. The figure shows that PAG is suppressed, while the expression of PAG-YFP (the resistant molecule) is unaffected. Actin was included as a loading control. The cells were then either left untreated or stimulated for 5 minutes with anti-CD3 (C305). Cell lysates were immunoblotted to analyze proximal signaling as indicated. Actin staining was included as a loading control. Quantification can be found in the supplemental figure. (**D**) Jurkat T cells were prepared as described in (**A**) and fractions 2–4 from sucrose density gradients pooled, separated by SDS-PAGE, blotted, and probed as indicated. Each panel is representative of at least three independent experiments. The phosphotyrosine blot in (**B**) was originally published in Blood: Smida, *et al*. 2007 [15]. Quantification can be found in the supplemental material (Additional file 2: Figure S2).

To determine the function of PAG, the Jurkat T cell line was transfected with plasmids encoding PAG shRNA and the kinetic of PAG suppression monitored by Western blotting (Additional file 1: Figure S1). Upon PAG suppression, we observed an increase in the basal kinase activity of both Fyn and Lck, as measured by *in vitro* kinase assays (IVKs) (Figure 1A); however only the increase in Fyn appears significant (Additional file 2: Figure S2A). PAG-suppression also resulted in a dramatic increase in basal tyrosine phosphorylation, which was further enhanced upon TCR cross-linking (Figure 1B). The augmented basal tyrosine phosphorylation and enhanced Src kinase activity correlate well with previous reports that PAG is a negative regulator of Src kinases [4,5,10]. Further analysis using phospho-specific reagents demonstrated that in the absence of PAG the phosphorylation of the activatory tyrosine residues of the Src kinases, ZAP-70, and PLCγ1 were also enhanced (Figure 1B and Additional file 2: Figure S2B). We previously showed that PAG negatively regulates Ras by recruiting p120 RasGAP into the lipid rafts and that upon PAG-suppression, Ras activity increases 5-fold [15]. Notably, the expression of Csk and RasGAP are unaffected by the loss of PAG, indicating that although these negative regulators are present, in the absence of PAG they are no longer functional. To demonstrate that the effect we observe is due to the loss of PAG, we re-expressed a resistant PAG molecule and show that the phenotype can be reverted (Figure 1C). PAG-suppression results in an enhanced basal tyrosine phosphorylation as well as a basal activation of proximal signaling molecules, such as pZAP70 and pPLCγ. This is clearly reduced by the expression of the resistant PAG-YFP protein (lane 3) as indicated by the filled arrows (also Additional file 2: Figure S2C). The level of inducible phosphorylation (lane 4) is comparable to that seen in control cells (lane 6). Since PAG over-expression is inhibitory to T cells [4,10], it is not surprising that the induction of pPLCγ upon CD3 stimulation is clearly reduced (lane 2 versus lane 6) as indicated by the open arrows (also Additional file 2: Figure S2C). To investigate the mechanism behind the enhanced Src kinase activity, we analyzed the distribution of Csk using sucrose density gradients, since PAG was initially proposed to function as a negative regulator of Src kinases by recruiting Csk into the lipid rafts [4] (Figure 1D). Indeed, reduced PAG expression results in a 50% decrease in Csk recruitment to the lipid rafts, consistent with this regulatory mechanism. The recruitment of LAT appears slightly increased (Additional file 2: Figure S2D).

### PAG suppression induces T-cell unresponsiveness

**Figure 2:**
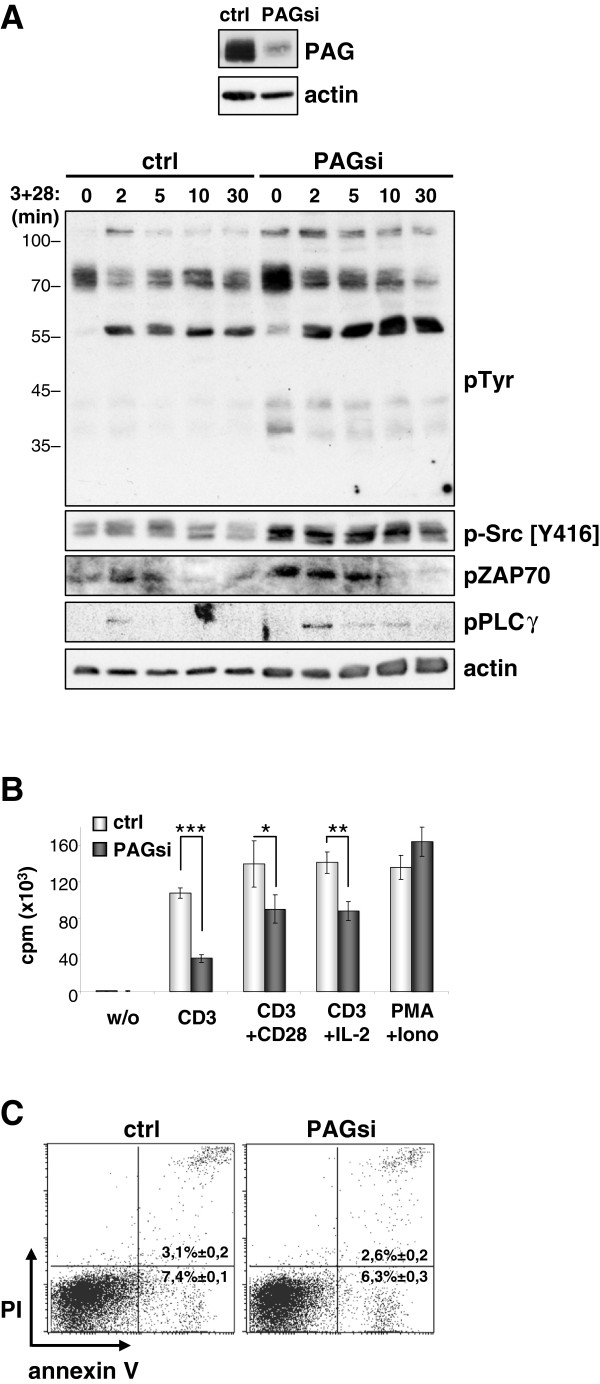
**Decreased proliferation in primary human T cells.** (**A**) Primary human T cells were transfected with siRNA oligonucleotide targeted against Renilla (ctrl) or PAG (PAGsi). Three days later, the cells were stimulated through CD3 plus CD28, lysed and immunoblotted with the indicated antibodies. Actin staining is shown as a loading control. The inset box shows the efficiency of PAG downregulation. (**B**) Primary human T cells were transfected with either Renilla siRNA (ctrl) or PAG siRNA (PAGsi) oligonucleotides and left for three days to downregulate PAG. Then, the cells were inoculated on a plastic plate coated with anti-CD3, anti-CD3+anti-CD28, anti-CD3 plus addition of exogenous IL-2 or uncoated (w/o). The incorporation of ^3^H-Thymidine was measured at 72 hours and is shown as the mean value of triplicates (±SD). Data were analyzed by the Student’s t-test (*, P<0.05; **, P<0.01; ***, P<0.001). (**C**) The cells transfected as in (**B**) were stimulated on an anti-CD3+anti-CD28 coated plastic plate for three days and the cell survival was assessed by annexin V-FITC and PI staining. Data are representative of three independent experiments. The phosphotyrosine blot in (**A**) was originally published in Blood: Smida, *et al*. 2007 [15]. Quantification of the blots can be found in the supplemental material (Additional file 3: Figure S3).

We next investigated freshly isolated human peripheral blood T cells. Upon PAG suppression, we observed a similar trend showing enhanced proximal signaling (Figure 2A and Additional file 3: Figure S3). We next looked to see whether these enhanced proximal signals also translated into enhanced functional responses. Surprisingly however, cells lacking PAG proliferated less well than the control transfected cells (Additional file 2: Figure 2B). This is in contrast to *Pag1*-deficient murine T cells, which proliferated equally as well as wild type cells [20,21]. Previously, we have shown that primary murine T cells receiving a strong stimulus (such as TCR cross-linking with soluble antibodies) undergo apoptosis [25]. Therefore we reckoned that the enhanced proximal signaling we observe was actually being translated into a death-inducing stimulus. However, further analysis showed no induction of apoptosis (Figure 2C) and no increase in caspase 3 activity or alteration in FasL expression (Additional file 4: Figure S4A and S4B respectively). This led us to conclude that the enhanced proximal signals were not instructing these cells to die.

**Figure 3:**
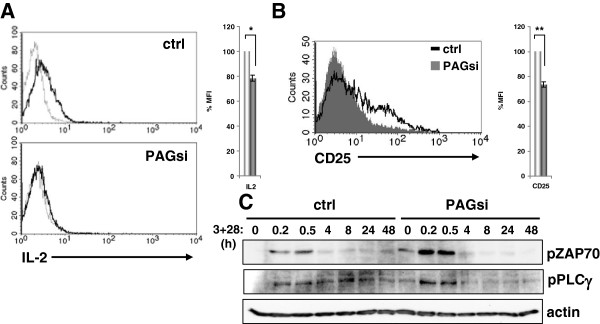
**PAG down-regulation results in unresponsiveness.** (**A**) Primary human T cells transfected either with Renilla (ctrl) or PAG (PAGsi) siRNA were stimulated on an anti-CD3 coated plastic plate for 24 hours and the production of IL-2 was assessed by intracellular FACS staining. (**B**) The cells treated as in (**A**) were used to determine the upregulation of surface CD25 expression by flow cytometry. In the histograms, white bars represent ctrl, grey bars PAGsi. (*, P<0.05; **, P<0.01) (**C**) Primary human T cells transfected either with Renilla (ctrl) or PAG (PAGsi) siRNA were stimulated with CD3+CD28 antibodies for the indicated time, lysed and subjected to Western blotting. The activation of ZAP70 and PLCγ was determined by phospho-specific antibodies. Actin staining is shown for equal loading. Data are representative of three (**A**, **B**) or two (**C**) independent experiments. Quantification is shown in the supplemental material (Additional file 6: Figure S5).

However, death is not the only response that a T cell can give upon too strong stimulation. Further analysis of PAG-suppression showed that these T cells clearly possess a defect in IL-2 production (Figure 3A) as well as an inability to up regulate CD25/IL-2Rα expression (Figure 3B). The inability of PAG-suppressed T cells to upregulate the IL-2R may explain why the addition of exogenous IL-2 did not rescue proliferation (Figure 2B). Together, these data led us to conclude that instead of directing T cells to die, the loss of PAG was actually instructing them to become unresponsive.

### T-cell unresponsiveness is not due to cell-cycle arrest or protein degradation

A number of mechanisms are known that lead to T-cell unresponsiveness. One such mechanism is the induction of cellular senescence via p53 activation, which induces cell-cycle arrest and thereby prevents activated oncogenes from inducing tumorigenesis [26]. We reasoned that since both Src kinases and Ras are known oncogenes and we observe enhanced activity in these enzymes, cell-cycle arrest might be induced. However, we observed neither an increase in the level of active p53, nor a decrease in pFOXO, which regulates cell cycle progression via p27. Moreover, we found reduced expression of the cell cycle inhibitors p21 and p53 in the nuclear extracts of PAG-suppressed cells (Additional file 5: Figure S6A), leaving us to conclude that an induction of cell-cycle arrest was not the mechanism responsible for unresponsiveness.

Alternatively, we hypothesized that the increased Src kinase activity in PAG-knockdown cells might also lead to the activation of other negative regulators that could suppress signaling. Indeed, when we extended our kinetic analysis of ZAP-70 and PLCγ activation (Figure 3C), we observed that control cells demonstrated a sustained activation, particularly of PLCγ (Additional file 6: Figure S5). In contrast, PAG-suppressed cells initially showed an enhanced response (similar to that seen in Figures 1B and 2A) that was abruptly truncated suggesting that a negative regulatory pathway had indeed been activated.

As Fyn is thought to induce negative regulatory pathways in T cells [27], we investigated a known substrate of Fyn, the E3 ubiquitin ligase Cbl. Within T cells, Cbl is known to target several important signaling components for degradation, which results in an abrogation of T-cell signaling. Although we observed an increase in phospho-Cbl upon PAG down-regulation, we saw no alteration in the expression of Lck or ZAP-70, two well characterized Cbl substrates [25,28] (Additional file 5: Figure S6B), suggesting that this mechanism was also not enabled.

### PAG suppression induces CTLA-4 hyper-phosphorylation and Shp-1 recruitment

One of the best characterized negative regulators of T cells is the co-stimulatory molecule CTLA-4 (cytotoxic T lymphocyte antigen 4). The endocytic adaptor AP-1 sequesters CTLA-4 within the Golgi. However, upon T-cell activation, tyrosine phosphorylation of CTLA-4 displaces AP-1, resulting in an increased surface expression and the subsequent recruitment of inhibitory molecules, such as the phosphatases Shp-2 and/or PP2A (reviewed in [29-31]).

**Figure 4:**
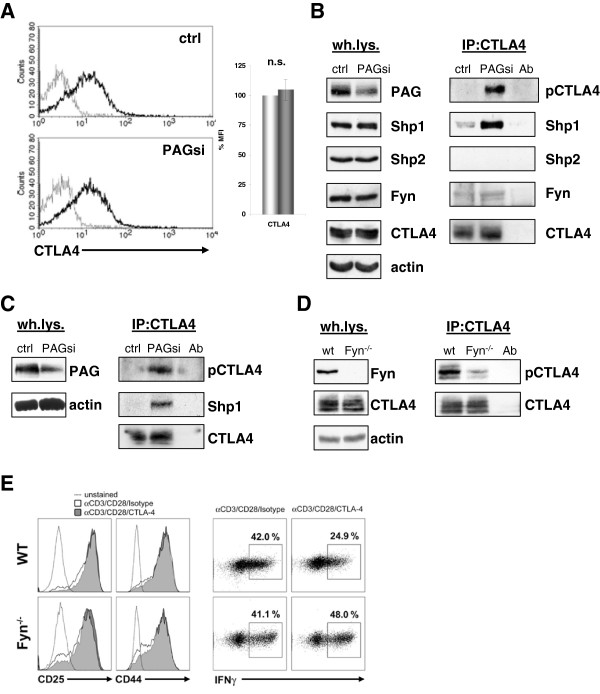
**Enhanced Src kinase activity results in phosphorylation of CTLA-4.** (**A**) Primary human T cells transfected with Renilla (ctrl) or PAG (PAGsi) siRNA were stimulated for 72 hours on an anti-CD3+anti-CD28 coated plastic plate. The surface expression of CTLA-4 was measured by extracellular FACS staining. (**B**) T cells transfected as in (**A**) were stimulated overnight on anti-CD3+anti-CD28 coated plastic plate, lysed and CTLA-4 was immunoprecipitated. Samples were immunoblotted for phospho-tyrosine (4G10), Shp1, Shp2, Fyn, and CTLA-4. Antibody control (Ab) was included in the IP’s. Panel left shows whole lysates. Data are representative of three independent experiments. Quantification of the blots can be found in the supplemental material (Additional file 7: Figure S7). (**C**) To exclude an off-target effect of our siRNA, a pool of three siRNAs (all differ from our sequence) was used for the same experiment as in (**B**). (**D**) Murine T cells from wildtype and Fyn knockout mice were cultured for three days on anti-CD3+anti-CD28 coated plastic plate, lysed and CTLA-4 was immunoprecipitated. Western blot of a phospho-tyrosine (4G10) and total CTLA-4 staining is shown. Panel left shows whole lysates. Data are representative of two independent experiments. (**E**). The CTLA-4 mediated reduction of IFNγ production is abbrogated in *Fyn*^−/−^ mice. Naïve (CD62L^high^) CD8^+^ T cells from WT or Fyn^−/−^ mice were stimulated with anti-CD3/CD28/CTLA-4 or anti-CD3/CD28/Isotype-coupled microspheres. Surface expression of the activation markers CD25 and CD44, as well as intracellular IFNγ expression were detected by flow cytometry after 2 days of activation.

Our analysis of PAG-suppressed primary human T cells showed no observable difference in either the level of surface or total CTLA-4 expression (Figure 4A and 4B). However, when we isolated CTLA-4 by immunoprecipitation, we found that PAG-suppression resulted in a dramatic enhancement in tyrosine phosphorylation compared to control transfected cells, where CTLA-4 phosphorylation is barely detectable (Figure 4B). This was not due to off-target effects, as we observed exactly the same phenotype with the multiplicity control; siRNA oligos targeting other sequences within the PAG molecule (Figure 4C). Surprisingly, the enhanced phosphorylation of CTLA-4 resulted in a substantial recruitment of the phosphatase Shp-1 (Figure 4B, Figure 4C and Additional file 7: Figure S7), whereas Shp-2 was undetectable. Like PAG, CTLA4 is also reported to function from lipid rafts [32-34]; a membrane compartment that is important for propagating TCR signals. Thus, although the suppression of PAG expression resulted in enhanced proximal signaling, it fails to induce functional T-cell responses possibly due to the induction of an auxiliary negative feedback loop involving CTLA-4-associated Shp-1. To test the validity of this mechanism, we next looked to see whether Fyn, which shows increased activity after PAG knockdown, was indeed responsible for phosphorylating CTLA-4 in primary T cells. Previous reports have demonstrated by co-expression that a number of Src kinases (such as Fyn, Lyn, or Lck) are able to phosphorylate CTLA-4 [35-37]. In support of this mechanism, we also found that Fyn associated with CTLA-4 (Figure 4B) in primary T cells, as had previously only been shown by co-expression [35,36]. When we immunoprecipitated CTLA-4 from activated murine T cells (Figure 4D), we found that CTLA-4 phosphorylation was indeed dramatically reduced in the absence of Fyn. Since phosphorylation is thought to be important for CTLA-4 function, we next tested whether CTLA4 is functional in *Fyn*-deficient mice. Naive CD8^+^ T cells were activated with immobilized antibodies to CD3 and CD28 in the presence or absence of CTLA-4 cross-linking. Based upon the upregulation of CD25 and CD44, all cells appear to have been activated (Figure 4E left panel). As expected, cross-linking of CTLA-4 inhibits the ability of WT cells to produce IFNγ [38], whereas this inhibitory function is lost in the absence of Fyn (Figure 4E right panel). These results support our model that the enhanced Fyn activity observed upon PAG suppression can activate alternative feedback mechanisms mediated by CTLA-4 and further supports the role of Fyn as a negative regulatory kinase in T-cell signaling [27].

### Co-suppression of CTLA-4 restores proliferation in PAG-suppressed cells

**Figure 5:**
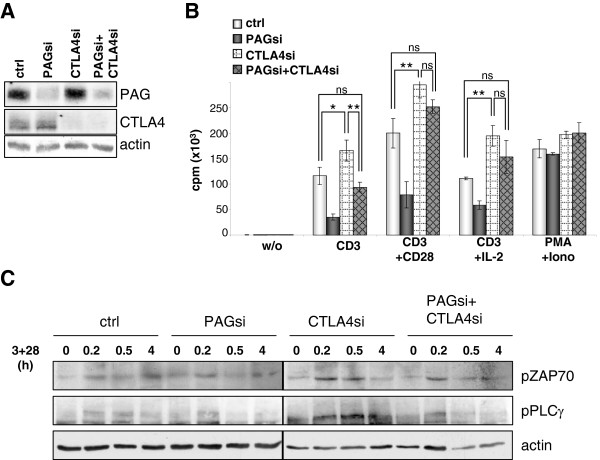
**CTLA-4 suppression restores the defect in proliferation caused by PAG suppression.** Primary human T cells were transfected with siRNAs targeting Renilla (ctrl), PAG (PAGsi), CTLA-4 (CTLA4si) or PAG + CTLA-4 (PAGsi+CTLA4si). Cells were then stimulated with indicated stimuli for three days on a plastic plate. (**A**) To demonstrate the downregulation of protein expression by siRNAs, total cell lysate staining for PAG and CTLA-4 is shown. (**B**) The proliferation was measured by ^3^H-Thymidin incorporation at 72 hours. Mean value of triplicates (±SD) is depicted. Data were analyzed by the Student’s t-test (*, P<0.05; **, P<0.01). Data are representative of three independent experiments. (**C**) Primary human T cells transfected with the siRNAs indicated were stimulated with CD3 antibodies for the indicated time, lysed and subjected to Western blotting. The activation of ZAP70 and PLCg was determined by phospho-specific antibodies. Actin is shown for equal loading. Quantification of the blots can be found in the supplemental material (Additional file 8: Figure S8).

The final question was whether removing CTLA-4 is sufficient to negate the effect of PAG suppression and restore proliferation. Therefore, we compared the proliferative capacity of control and PAG-suppressed T cells, both with and without additional CTLA-4 suppression. Figure 5A shows that the siRNA was effective at suppressing protein expression for both PAG and CTLA-4. Figure 5B shows that in the absence of PAG, T cells became unresponsive (as shown in Figure 2B), while in the absence of CTLA-4, T cells became hyper-proliferative. Interestingly, for T cells in which both PAG and CTLA-4 were co-suppressed, the proliferative capacity was equal to or better than that observed for the control transfected cells, indicating that the suppressive mechanism induced by PAG-suppression is negated by removing CTLA-4. A similar trend was also seen when we analyzed the proximal signaling in these cells. Most strikingly, the suppression of CTLA-4 alone resulted in an enhanced activation of PLCγ, which appears to be reverted upon the removal of PAG (Figure 5C and Additional file 8: Figure S8). The removal of PAG and CTLA-4 counteract one another both functionally and biochemically suggesting that both molecules regulate the same pathway.

## Discussion

**Figure 6:**
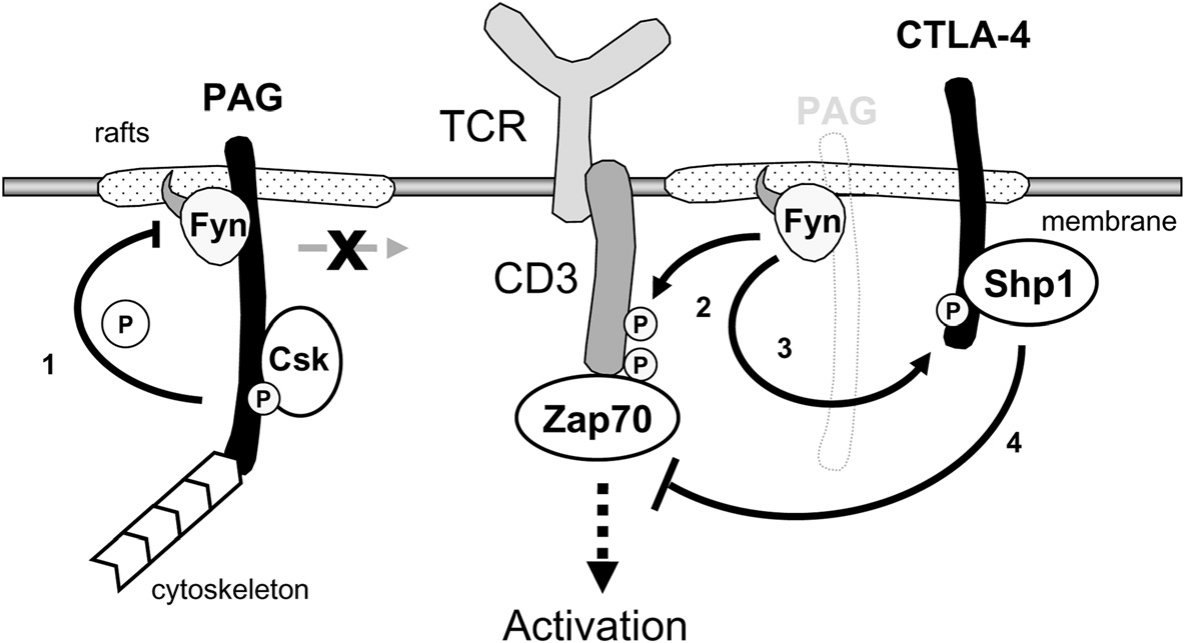
**Model of CTLA-4 induced T-cell unresponsiveness. 1**. In resting T cells (left panel) PAG restricts mobility of the lipid rafts and recruits Csk to the membrane, where it blocks activation of the Src family kinases. **2**. In the absence of PAG (right panel) Fyn activity is increased, resulting in enhanced proximal signaling events. **3**. However, at a later time point enhanced Fyn activity also leads to hyper-phosphorylation of CTLA-4. **4**. Phosphorylated CTLA-4 can recruit the protein tyrosine phosphatase Shp-1 to the lipid rafts, where Shp-1 blocks T-cell activation by inactivating ZAP-70.

Here, we verify the role of PAG as a negative regulator of Src kinases. Additionally, in the absence of PAG, we identify a fail-safe mechanism that induces T-cell unresponsiveness via a Fyn-dependent phosphorylation of CTLA-4 (Figure 6). Consistent with the negative regulatory function of PAG, we observe both enhanced Src kinase activity and increased basal tyrosine phosphorylation upon PAG suppression; a phenotype that could be reverted by re-expressing a resistant PAG molecule (Figure 1C). Interestingly, the phosphotyrosine profiles (Figures 1B and 2A) show that the transmembrane adaptor LAT (pp36/38) was also constitutively phosphorylated in resting PAG-suppressed T cells, indicating that the enhanced phosphorylation was not restricted to direct substrates of the Src family kinases.

A similar phenotype of enhanced Src kinase activity was observed upon Csk suppression [39]. However, the secretion of IL-2 was enhanced in primary human T cells rather than inhibited, as reported here. The differences observed between PAG and Csk downregulation may be due to Csk-independent functions of PAG for Src kinase regulation. For Src, an SH2-domain-dependent interaction with PAG was shown, which resulted in the sequestration of Src into lipid rafts away from non-raft Src substrates, inhibition of downstream activation of MEK, ERK and Akt and suppression of Src-mediated oncogenic transformation in the absence of Csk. Similarly, in these Csk−/− cells a palmitoylation mutant of Fyn showed redistribution away from lipid rafts and enhanced kinase activity, demonstrating that lipid raft localisation inhibits Fyn activity in the absence of Csk and independent of the presence of the negative-regulatory tyrosine Y529 [13]. Our results here show that downregulation of PAG releases this inhibition. Despite unchanged raft-association of Fyn its activity is now increased. Notably, despite the enhancement of proximal TCR signaling, PAG suppression had no effect upon ERK activation or calcium-flux (Additional file 9: Figure S9). This may be due to the fact that activation of these molecules is already maximal and therefore cannot be further enhanced or that there is a spatial segregation of activators and effectors. However, despite our inability to influence these molecules, PAG-suppressed T cells still became unresponsive due to a hyper-phosphorylation of CTLA-4. Here, we show for the first time that Fyn is largely responsible for CTLA-4 phosphorylation and function in primary T cells. These observations are supported by previous studies using co-expression to show that Fyn phosphorylates CTLA-4 [35-37] and also support the idea that Fyn primarily contributes to negative regulation in T cells [27].

One function of CTLA-4 is to reverse the TCR-induced stop signal [40]. In line with this activity, we previously reported that PAG-suppression resulted in both an enhanced spontaneous and specific migration of T cells [24].

To our knowledge, this is the first report demonstrating Shp-1 recruitment to CTLA-4. This fits with previous observations that CTLA-4 functions from the lipid rafts [32-34] and targeting Shp-1 to the rafts blocks T-cell activation [41]. In this context, Shp-1 could induce unresponsiveness by inactivating key signaling molecules such as Lck, ZAP-70, and PLCγ. Indeed, CTLA-4 has previously been shown to inhibit phosphorylation of ZAP-70 at Y319 [42] and in the absence of CTLA-4 we observe enhanced phosphorylation of both ZAP70 and PLCγ (Figure 5C and Additional file 8: Figure S8). Similarly, the phosphorylation of ZAP-70 and PLCγ was abruptly truncated following activation of PAG-suppressed cells (Figure 3C). Previously, Shp-1 was shown to bind to phosphorylated peptides corresponding to the cytoplasmic tail of CTLA-4 [42,43], however an involvement of Shp-1 was ruled out, as CTLA-4 was shown to be inhibitory in moth-eaten mice [44,45]. Given that CTLA-4 binds Shp-2 in mice [46], one could attribute this differential recruitment to either redundancy or species-specific differences, as shown for LAIR-1 (leukocyte-associated Ig-like receptor-1) [47,48].

Despite the hyper-phosphorylation of CTLA-4, we observed no detectable increase in the levels of surface CTLA-4 expression (Figure 4A). This was somewhat surprising, as CTLA-4 expression should be regulated by phosphorylation of the YVKM motif within the cytoplasmic tail, which prevents binding of AP-2 and thereby blocks endocytosis of the receptor [reviewed in [29-31]. However, Hu *et al.* (2001) have previously shown that constitutively active Lck, or Fyn, phosphorylate CTLA-4 within the Golgi [37]. This supports our hypothesis that pCTLA-4 may not need to be at the surface to function and suggests that additional mechanisms besides phosphorylation may regulate surface expression. An alternative interpretation may be that the enhanced phosphorylation of CTLA4 we observe also increases the rate of internalization, such that there is no observable change in surface expression.

While co-stimulation of CTLA-4 is known to inhibit T-cell activation, it is interesting to consider that the inhibitory effect of CTLA-4 observed here is independent of ligand, as the cells were stimulated with plate-bound antibody and not antigen presenting cells. This is in agreement with previous reports demonstrating ligand-independent inhibitory functions for CTLA-4. First, mice express a ligand-independent isoform of CTLA-4 that generates inhibitory signals [49,50]. Additionally, the expression of a ligand-nonbinding CTLA-4 was sufficient to inhibit T-cell proliferation and block cytokine production on an otherwise CTLA-4-deficient background [51]. Also, recent reports have shown that the cytoplasmic domain of CTLA-4 (ctCTLA-4) alone is sufficient to inhibit T-cell activation and prevent autoimmunity [52-54]. Taken together, these studies suggest that competing with CD28 for ligand is only one of several inhibitory mechanisms utilized by CTLA-4 and support the hypothesis that there is an intrinsic inhibitory nature to CTLA-4. Since the majority of CTLA-4 is intracellular, with only a minor fraction of molecules (~10%) appearing on the cell surface [55], we consider it likely that these molecules also have a function. The inhibitory nature of CTLA-4 is further supported by the hyper-proliferative capacity of CTLA-4-suppressed T cells to plate-bound antibody (Figure 5), which agrees with previous reports showing that blocking CTLA-4 enhances proliferation [56,57]. This mechanism is supported by studies demonstrating the presence of intracellular CTLA-4 in resting primary human T cells [41,58]. Taken together, the data suggest that the mere presence of CTLA-4 is inhibitory to T cells, as was recently shown for PD-1 [59]. A similar inhibitory role has been proposed for Shp-1 [44,60], and a possible contribution of CTLA-4 to setting the activation threshold within T cells has been suggested for primed TCR-transgenic cells [61]. This intrinsic inhibitory mechanism may also contribute to the unresponsive nature of regulatory T cells, which constitutively express CTLA-4 [60,62].

## Conclusion

We demonstrate that PAG is indeed a negative regulator in T cells and show that the loss of PAG alone is not sufficient to induce lymphocyte transformation. Instead, the enhanced Src kinase activity induced by the loss of PAG triggers an auxiliary negative feedback loop involving CTLA-4. Thus, it appears that multiple mechanisms have evolved to ensure a tight regulation of T-cells. Indeed, the mechanism identified here may explain T-cell unresponsiveness in older mice, which show decreased PAG expression, enhanced proximal signaling, and defective proliferation [63]. Further analysis has also shown that aged mice upregulate the expression of inhibitory co-stimulatory molecules, such as CTLA-4 and PD-1 [64]. The importance of this mechanism for inducing T-cell unresponsiveness may now take on a broader context, as PAG expression has been shown to be suppressed by epigenetic histone modification [65]. Indeed, data generated by the Immunological Genome project [http://www.immgen.org] suggest that within the population of αβ T cells, *Pag1* expression is lowest in tumor-infiltrating CD8^+^ and splenic CD4^+^CD25^+^Foxp3^+^ regulatory T cells, both of which are CTLA-4^+^.

The loss of PAG has also revealed a novel contribution of CTLA-4 to setting the activation threshold within T cells. This suggests that in addition to lacking functional regulatory T cells, the CTLA-4 knockout mice also possess a lower threshold for activation, which may contribute to the hyper-proliferative syndrome that leads to autoimmunity in these mice [29,30,46]. Indeed *in vitro* stimulation of T cells from *Ctla4*-deficient mice showed an enhanced expression of CD69 per cell [66] indicative of stronger TCR signaling.

## Methods

### Antibodies and reagents

The following hybridoma supernatants were produced within our Institute: mouse anti-phosphotyrosine (clone 4G10), mouse anti-CD3ε (clones OKT-3 (IgG) and MEM-92 (IgM)), mouse anti-TCRβ (clone C305, IgM), mouse anti-CD28 (clone 248.23.2, IgM), and mouse anti-PAG (clone MEM-255). Mouse anti-Fyn-02 was kindly provided by Dr. Vaclav Horejsi. Rabbit anti-phospho-Src (pY418), mouse anti-Lck (3A5), rabbit anti-Lck and rabbit anti-Fyn were purchased from BioSource, mouse anti-Pan-Ras (Ab-4) from Oncogene, mouse anti-RasGAP (B4F8), mouse anti-human CTLA-4 (BNI3), hamster anti-mouse CTLA-4 (UC10-4 F10-11), anti-mouse CD8 (196), anti-mouse CD25 (7D4), anti-mouse CD44 (IM7), anti-mouse CD62L (MEL14), anti-mouse IFNγ (AN18.17.24) were from BD Biosciences, goat anti-CTLA-4 (C-19), rabbit anti-Shp1 (C19), mouse anti-Shp2 (B1), rabbit anti-GFP/YFP (FL), and rabbit anti-Csk (C20) from Santa Cruz. Phospho-specific antibodies against p-ZAP70 (pY319) and p-PLCγ1 (pY783) were obtained from Cell Signaling Technology. Secondary antibodies goat-anti-mouse-HRP and goat-anti-rabbit-HRP were obtained from Dianova, donkey-anti-goat-HRP from Santa Cruz. Phorbol myristate acetate (PMA) and N-dodecyl β-D-maltoside (lauryl maltoside (LM)) were purchased from Calbiochem. Igepal (Nonidet P-40 (NP-40)), ionomycin, glutathione-sepharose, GDP and mouse anti-β-actin (AC-15) were from Sigma. Human recombinant interleukin 2 (IL-2) was purchased from Tebu-bio. The PAG-YFP vector was kindly provided by Dr. Shigeyuki Nada.

### Cell cultures

Approval for these studies was obtained from the Ethics Committee of the Medical Faculty at the Otto-von-Guericke University, Magdeburg, Germany. Informed written consent was obtained in accordance with the Declaration of Helsinki. Human T cells were prepared as previously described [15]. The Jurkat E6.1 T cell line was cultured as described [24].

### Mouse experiments

Fyn knockout mice were kindly provided by Dr. Rose Zamoyska and maintained under pathogen-free conditions. Experiments were performed according to the guidelines of the state of Sachsen-Anhalt. Splenic T cells were purified by non-T cell depletion using a Pan T cell isolation Kit [Miltenyi Biotec]. Purified T cells were cultured for three days on 24-well-plates coated with anti-CD3 (2C11; 10 μg/ml) plus anti-CD28 (1 μg/ml) [both BD]. 20x10^6^ mouse T cells were used for immunoprecipitation with hamster anti-mouse CTLA-4 as described for human T cells below.

Alternatively, crosslinking of CTLA-4 (CD152) on CD8 cells was performed using latex microspheres coated with antibodies. In brief, 10^7^ microspheres/ml were suspended in PBS with CD3 (0.75 μg/ml), CD28 (2.5 μg/ml), CTLA-4 or a hamster control antibody (A19-3, 8 μg/ml) and incubated for 1 h at 37°C, followed by washing in PBS and blocking with complete media. CD8 T cells (1.5x10^6^/ml) were stimulated at a ratio of 1:1 with antibody-coupled microspheres. Specificity of crosslinking of CD152 with antibody-coupled microspheres was controlled by stimulating naive CD8 T cells (CD8^+^ CD62L^high^) of C57BL/6 OT-1 CTLA4^−/−^ mice with CD3, CD28, and CTLA-4 or CD3, CD28, and isotype-coupled microspheres for 2d followed by analyzing expression of IFNγ as described below.

### RNA inhibition

For PAG siRNA, the human sequence 5^′^ GCGAUACAGACUCUCAACATT 3^′^ corresponding to Shima *et al*. [23] was either used as RNA oligos [Invitrogen] in primary human T cells or cloned as shRNA into the vector pCMS3-EGFP for Jurkat T cells. All constructs were sequenced to ensure integrity. To rule out off-target effects of our PAG siRNA oligonucleotide, some experiments were repeated with an siGENOME SMARTpool of 4 siRNAs purchased from Thermo Scientific. For CTLA-4 downregulation, a STEALTH pool of three siRNAs [Invitrogen] was used.

For transfection, primary human T-cells (8x10^6^) were washed with PBS, resuspended in 200 μl Optimem [Invitrogen] and transfected with siRNA in a 4 mm cuvette [BioRad] with a square-wave pulse using a Genepulser X-cell [BioRad] at 1000 V, 0.5 ms, 2 pulses, gap between pulses 5 s. Jurkat T cells were electroporated with 30 μg DNA in a 4 mm cuvette [BioRad] using Gene Pulser II [BioRad] at 210 V, 950 μF. Protein expression was monitored by Western blotting to determine the time required for optimal suppression.

### Cell stimulation

Primary human T cells were washed once with RPMI medium without FBS and stimulated with CD3 (MEM-92) plus CD28 antibody supernatants for indicated times at 37°C (100 μl per 5x10^6^ cells). Stimulation was stopped with 1 ml ice-cold TBS and the cells lysed immediately. Jurkat T cells were stimulated in the same way with anti-TCR (C305) plus anti-CD28 supernatants.

### Immunoprecipitation and western blotting

Cell lysates were prepared as previously described [15]. For CTLA-4 immunoprecipitation, 10x10^6^ activated T cells were lysed in 250 μl lysis buffer and immunoprecipitated with 1 mg/ml BSA, CTLA-4 antibody, and protein A sepharose for 2–18 h with rotation at 4°C as described [15].

### *In vitro* kinase assay

Cells (10x10^6^/sample) were lysed and immunoprecipitated with either Fyn-02 or rabbit anti-Lck and Protein A-Sepharose. *In vitro* kinase assays were performed as previously described [4].

### Proliferation assays

The incorporation of [3H]-thymidine (0.3 μCi/well, specific activity 50 Ci/mmol) [PerkinElmer] at the end of the three day stimulation was measured by liquid scintillation as previously described [15].

### Flow cytometry

Transfected primary human T cells were stimulated on a 24-well-plate coated with anti-CD3 or anti-CD3 plus anti-CD28 for indicated time, washed once with PBS and stained with CD25-FITC, FasL-PE or CTLA-4-PE [all BD] for 20 min on ice. After one wash with cold PBS, samples were analyzed on a FACS Calibur using the Cell Quest Pro software [BD].

For intracellular IL-2 staining, Brefeldin A [Calbiochem] was added at 1 μg/ml for the last 6 hours of culture and the cells were stained with IL-2-PE [Miltenyi Biotec] using the Inside Stain Kit [Miltenyi Biotec] according to the manufacturer’s instructions.

The activated caspase 3 within the stimulated cells was detected with the Caspase-3 Detection Kit [Calbiochem] according to the manufacturer’s instructions.

Intracellular staining of murine T cells for IFNγ production was performed using fixed cells. Fixation was performed by incubation of cells in 2% Formaldehyde (Merck) diluted in PBS for 20 min on ice. Permeabilization was achieved using 0.5% Saponin (Sigma) diluted in PBS/BSA.

### Apoptosis assay

Cell apoptosis was determined using the Annexin V-FITC Apoptosis Detection Kit [Bender MedSystems] according to the manufacturer’s instructions.

### Sucrose gradient centrifugation

Lipid raft fractionation was performed as previously described [4].

### Quantification

Films were scanned with an Epson Perfection 4990 Photo scanner and the optical density determined using Kodak 1D 3.6 software. The density of the band of interest was normalized using the loading control to the value in the control cells.

### Statistical analysis

All statistical analyses were performed using GraphPad Prism software. Comparison of two samples was performed using two-tailed Student’s t-test. One sample t-tests were used where indicated. Multiple samples were compared by a one-way ANOVA combined with a Tukey’s Multiple Comparison Post Test.

## Abbreviations

CBP: Csk binding protein; Csk: C-terminal Src kinase; CTLA-4: Cytotoxic T lymphocyte antigen 4; ERK1/2: Extracellular signal-regulated kinases 1/2; GAP: GTPase activating protein; IFNγ: Interferon gamma; IL-2: Interleukin 2; LAT: Linker for activation of T cells; PAG: Phosphoprotein associated with glycosphingolipid-enriched microdomains; PLCγ: Phospholipase C gamma; Shp1/2: Src homology phosphatase-1/2; shRNA: Small hairpin RNA; siRNA: Small inhibitory RNA; TCR: T cell receptor; YFP: Yellow fluorescent protein; ZAP-70: Zeta-associated protein of 70 kDa.

## Competing interests

The authors declare that they have no competing interests.

## Authors’ contributions

MSm designed and performed experiments, analyzed and interpreted results, and wrote the manuscript; CC, SG, NK, HL performed experiments and analyzed data; SL, LS analyzed data, interpreted results, and wrote the manuscript, MCB-W. Designed experiments, interpreted results, and wrote the manuscript, MSu contributed vital reagents; BS supervised the work, interpreted results, and edited the manuscript; and JAL directed the study, designed experiments, analyzed and interpreted results, and wrote the manuscript. All authors read and approved the final manuscript.

## Supplementary Material

Additional file 1: Figure S1 Kinetics of PAG suppression. Jurkat T cells were transfected with plasmids encoding PAG shRNA and the change in PAG expression monitored by Western blotting using the MEM-255 antibody. Actin staining is shown as a loading control. All samples were run on a single gel and blotted. The added line indicates where irrelevant samples were removed. Quantification of the data is provided in the histogram. Data were analyzed by the Student’s t-test (*, P<0.05; ***, P<0.001). Click here for file

Additional file 2: Figure S2 Quantification of Figure 1. **(A)** The autophosphorylation of Fyn and Lck in the kinase assays (IVK) (Figure 1A) were normalized with respect to the loading controls. The values for PAG siRNA was set to 1.0 and a One-Sample t test analysis was performed (shown is the mean ± SEM; *, P<0.05, n = 3). **(B)** The relative signal intensities of p-Src (pY416), p-ZAP-70, and p-PLCγ in Figure 1B were normalized with respect to the loading controls and the peak value set to 1.0 (the mean ± SEM is shown). Data are representative of p-ZAP-70 and p-PLCγ, n = 5, and pY416, n = 3 independent experiments. **(C)** The relative signal intensities of p-ZAP-70 and p-PLCγ in Figure 1C were normalized to the loading control. The peak value of the YFP-transfected control was set to 1.0 and the mean ± SEM is shown (*, P<0.05, n = 3). **(D)** The relative signal intensities of the blots for Csk and LAT in Figure 1D are shown. The data is normalized to the ctrl siRNA and the mean ± SEM is shown (n = 3). Grey bars (ctrl), black bars (PAGsi). The data have not been corrected for multiple testing. Click here for file

Additional file 3: Figure S3 Quantification of Figure 2. The relative signal intensities of p-Src (pY416), p-ZAP-70, and p-PLCγ in Figure 2A were normalized with respect to the loading controls and the peak value set to 1.0 (the mean ± SEM is shown). Data are representative of p-ZAP-70 and p-PLCγ, n = 3, and pY416, n = 2 independent experiments. Click here for file

Additional file 4: Figure S4 Strong signaling does not induce apoptosis. Primary human T cells transfected either with Renilla (ctrl) or PAG (PAGsi) siRNA were stimulated on an anti-CD3+anti-CD28 coated plastic plate for three days. (**A**) The activation of caspase 3 was determined using FITC-conjugated DEVD-FMK. Profiles of unstimulated (grey line) versus stimulated (black line) cells are shown. White bars (ctrl), grey bars (PAGsi). (**B**) The upregulation of FasL was analyzed by flow cytometry. Profiles of unstimulated (grey line) versus stimulated (black line) cells are shown. Data are representative of three independent experiments. Click here for file

Additional file 5: Figure S6 T-cell unresponsiveness is not due to oncogene-induced senescence or enhanced Cbl activity. **(A)** Enhanced SFK activity does not result in oncogene-induced senescence. Primary human T cells transfected with Renilla (ctrl) or PAG (PAGsi) siRNAs were stimulated for 72 hours on an anti-CD3+anti-CD28 coated plastic plate. Isolation of cytoplasmic and nuclear fractions was performed as previously described (10). Briefly, cells were resuspended in an hypotonic buffer and incubated with 10% NP-40. After centrifugation at 2000 rpm, 5 min, 4^o^C, the cytoplasmic fraction was obtained. Pellets were washed and lysed in a stringent lysis buffer for 1 hour at 4^o^C with agitation. Samples were then centrifuged at 13000 rpm, 10 min, 4^o^C and the supernatant was taken as the nuclear fraction. Both fractions were loaded on a 12% acrylamide gel and immunoblotted with phospho-p53, total p53, total p21 [all from Exbio] and pFOXO1 antibodies [Cell Signaling]. Lamin A [BioLegend] and GAPDH [Abcam] antibodies were used as markers to detect nuclear and cytoplasmic fraction respectively. Data are representative of three independent experiments. **(B)** PAG suppression enhances phospho-Cbl, but does not affect Lck or ZAP-70 expression. Primary human T cells transfected with Renilla (ctrl) or PAG (PAGsi) siRNAs were stimulated with anti-CD3+anti-CD28 for up to 24 hours, lysed and immunoblotted for phosphorylation of Cbl (pY731) [Cell Signaling] and total expression of ZAP-70 [BD] and Lck [Biosource]. Actin staining is shown as a loading control. Data are representative of two independent experiments. Click here for file

Additional file 6: Figure S5 Quantification of Figure 3. The relative signal intensities of p-ZAP-70 and p-PLCγ in Figure 3C were normalized to the loading control. The peak value of the control sample was set to 1.0. Data from two independent experiments are shown, in both cases the control samples show a sustained kinetic, whereas the signal in the PAG siRNA samples peaks earlier and terminates. Click here for file

Additional file 7: Figure S7 Quantification of Figure 4. The blots from the CTLA-4 immunoprecipitates were analyzed. The control values set to 1.0 and a One-Sample t test analysis was performed (shown is the mean ± SEM; *, P<0.05, n = 4). As shown in Figure 4, a significant increase in the phosphorylation of CTLA-4 and recruitment of Shp1 is observed, while the amount of Fyn and CTLA-4 remain unchanged. Click here for file

Additional file 8: Figure S8 Quantification of Figure 5. The relative signal intensities of p-ZAP-70 and p-PLCγ in Figure 5C were normalized with respect to the loading controls and the peak value set to 1.0 (the mean ± SEM is shown). Data are representative of at least three independent experiments. Click here for file

Additional file 9: Figure S9 PAG suppression does not affect TCR-induced ERK activation or calcium flux. (**A**) Jurkat T cells transfected either with control (ctrl) or PAGshRNA (PAGsi) constructs were stimulated with anti-CD3+anti-CD28 for the indicated time, lysed and immunoblotted for pERK [Cell Signaling]. Actin staining is shown for equal loading. Data are representative of three independent experiments. (**B**) Intracellular calcium flux was measured in primary human T cells transfected either with Renilla (ctrl; grey dashed line) or PAG siRNA (PAGsi, black solid line). Transfected T cells were washed with RPMI 1640 without phenol red and loaded with 5 μg/ml Indo-1 [Invitrogen] for 45 min at 37^o^C. The cells were washed briefly and incubated for an additional 30 min before measuring the FL4 (510/20 nm) versus FL5 (400/40 nm) ratio on an LSR1 flow cytometer [BD]. The cells were stimulated first with 50 μg CD3 (MEM-92) antibody supernatant and then with ionomycin (1 μg/ml). The addition of CD3 antibody and ionomycin is indicated by the filled and empty triangle, respectively. One representative experiment of three is shown. Click here for file
