# Supplementary figures and images for: PAG/Cbp suppression reveals a contribution of CTLA-4 to setting the activation threshold in T cells
Source: Cell Commun Signal. 2013 Apr 19;11:28. doi: 10.1186/1478-811X-11-28 (PMC3763844; doi:10.1186/1478-811X-11-28)

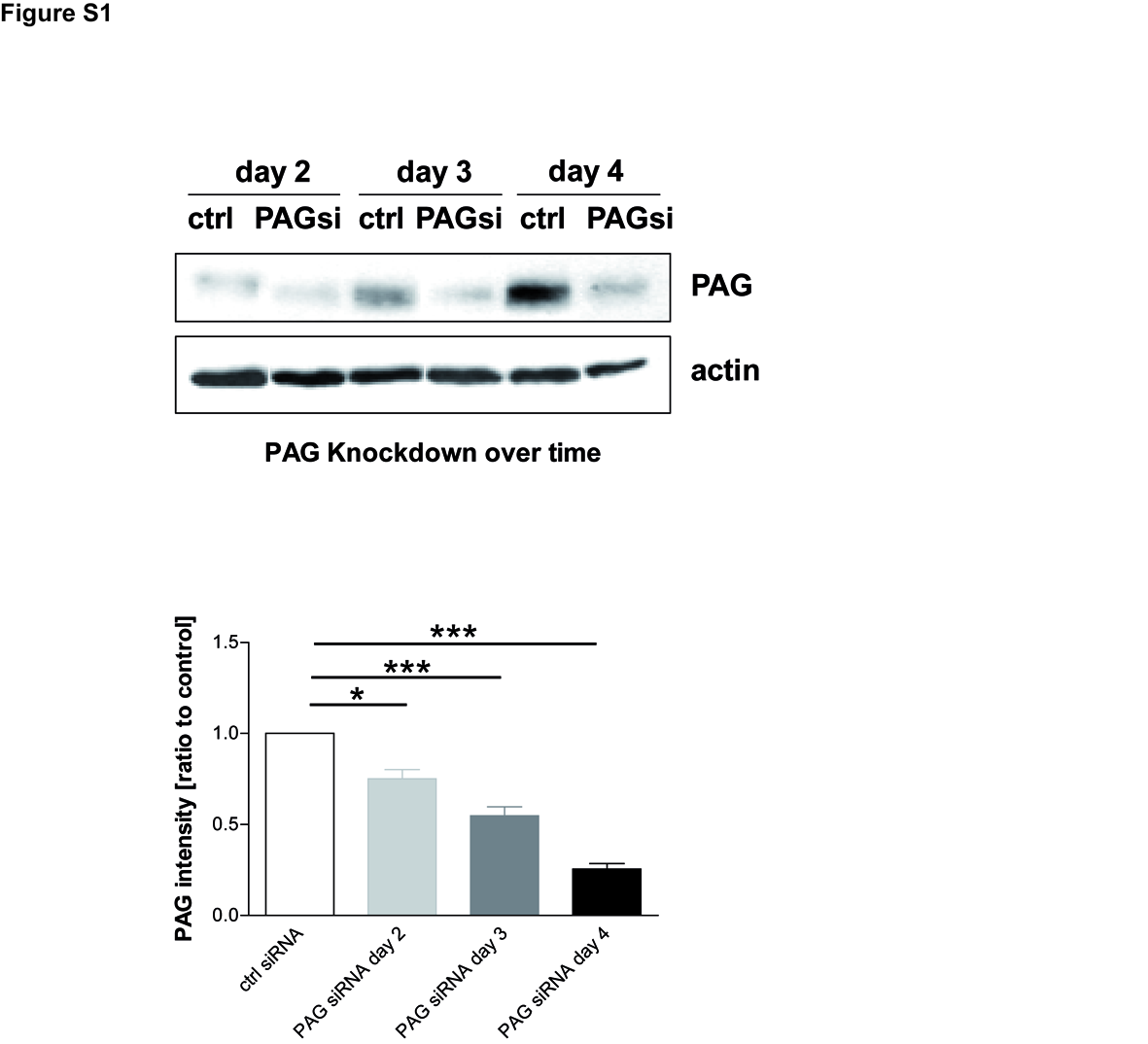

Supplement: Additional file 1: Figure S1 — Kinetics of PAG suppression. Jurkat T cells were transfected with plasmids encoding PAG shRNA and the change in PAG expression monitored by Western blotting using the MEM-255 antibody. Actin staining is shown as a loading control. All samples were run on a single gel and blotted. The added line indicates where irrelevant samples were removed. Quantification of the data is provided in the histogram. Data were analyzed by the Student’s t-test (*, P<0.05; ***, P<0.001). [file 1478-811X-11-28-S1.tiff]

**Figure S2**

**A**

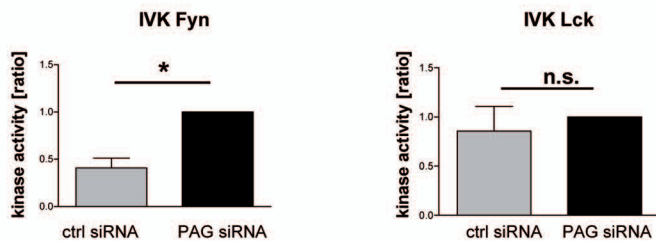

**B**

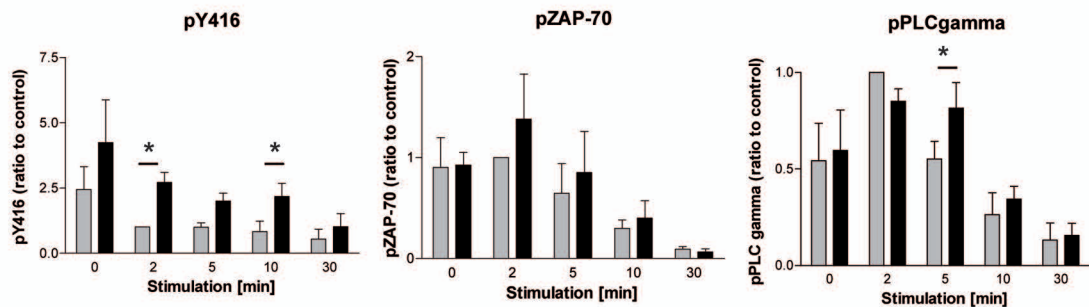

**C**

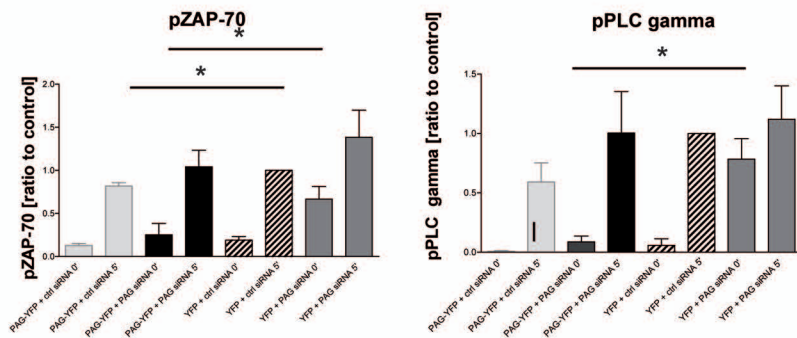

**D**

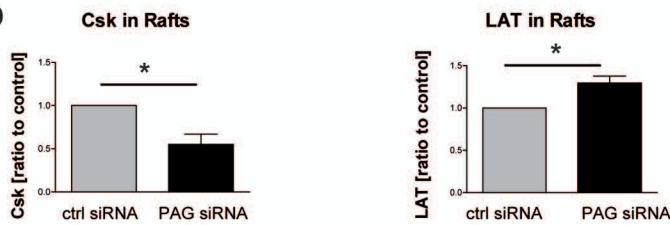

Supplement: Additional file 2: Figure S2 — Quantification of Figure 1. (A) The autophosphorylation of Fyn and Lck in the kinase assays (IVK) (Figure 1A) were normalized with respect to the loading controls. The values for PAG siRNA was set to 1.0 and a One-Sample t test analysis was performed (shown is the mean ± SEM; *, P<0.05, n = 3). (B) The relative signal intensities of p-Src (pY416), p-ZAP-70, and p-PLCγ in Figure 1B were normalized with respect to the loading controls and the peak value set to 1.0 (the mean ± SEM is shown). Data are representative of p-ZAP-70 and p-PLCγ, n = 5, and pY416, n = 3 independent experiments. (C) The relative signal intensities of p-ZAP-70 and p-PLCγ in Figure 1C were normalized to the loading control. The peak value of the YFP-transfected control was set to 1.0 and the mean ± SEM is shown (*, P<0.05, n = 3). (D) The relative signal intensities of the blots for Csk and LAT in Figure 1D are shown. The data is normalized to the ctrl siRNA and the mean ± SEM is shown (n = 3). Grey bars (ctrl), black bars (PAGsi). The data have not been corrected for multiple testing. [file 1478-811X-11-28-S2.pdf]

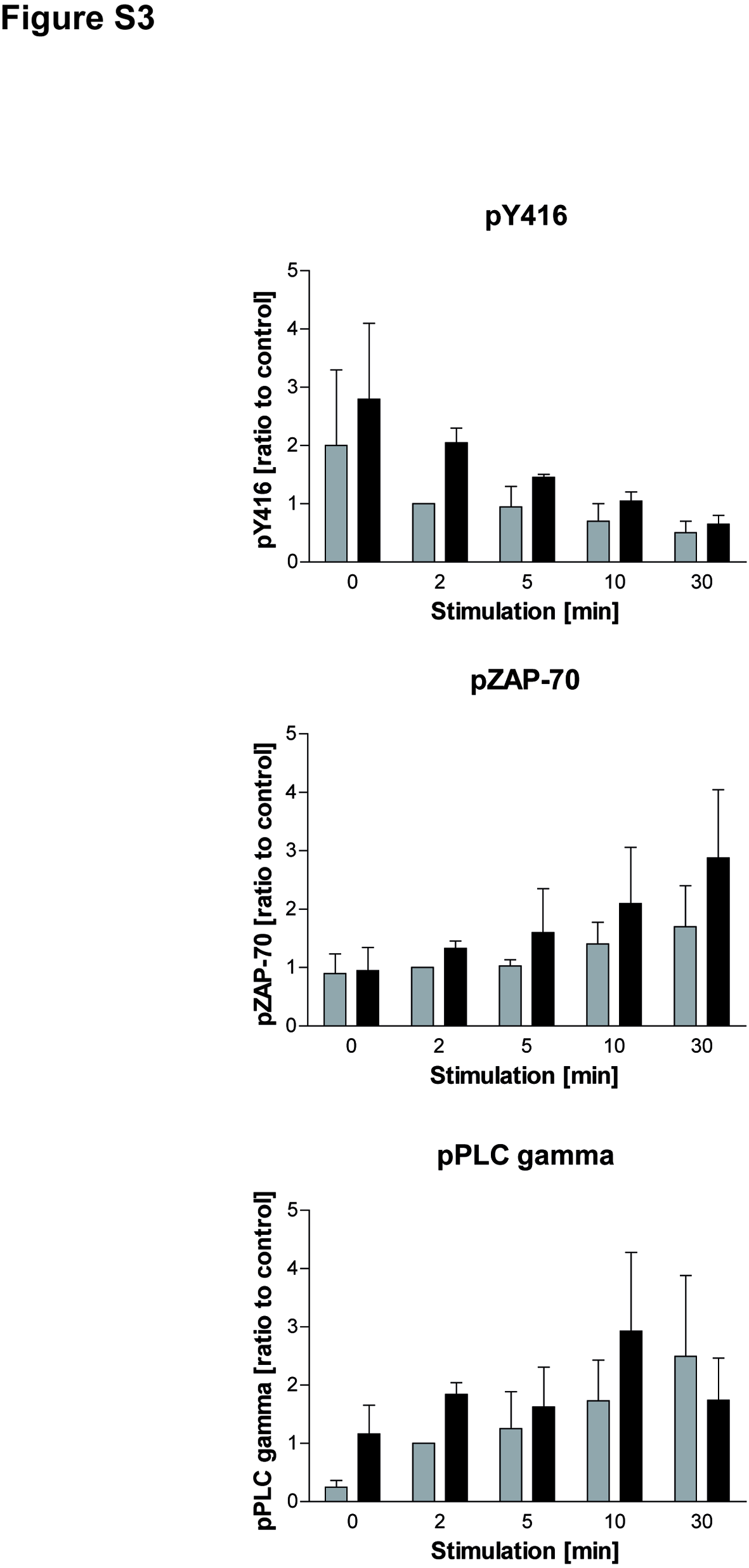

Supplement: Additional file 3: Figure S3 — Quantification of Figure 2. The relative signal intensities of p-Src (pY416), p-ZAP-70, and p-PLCγ in Figure 2A were normalized with respect to the loading controls and the peak value set to 1.0 (the mean ± SEM is shown). Data are representative of p-ZAP-70 and p-PLCγ, n = 3, and pY416, n = 2 independent experiments. [file 1478-811X-11-28-S3.tiff]

Figure S4

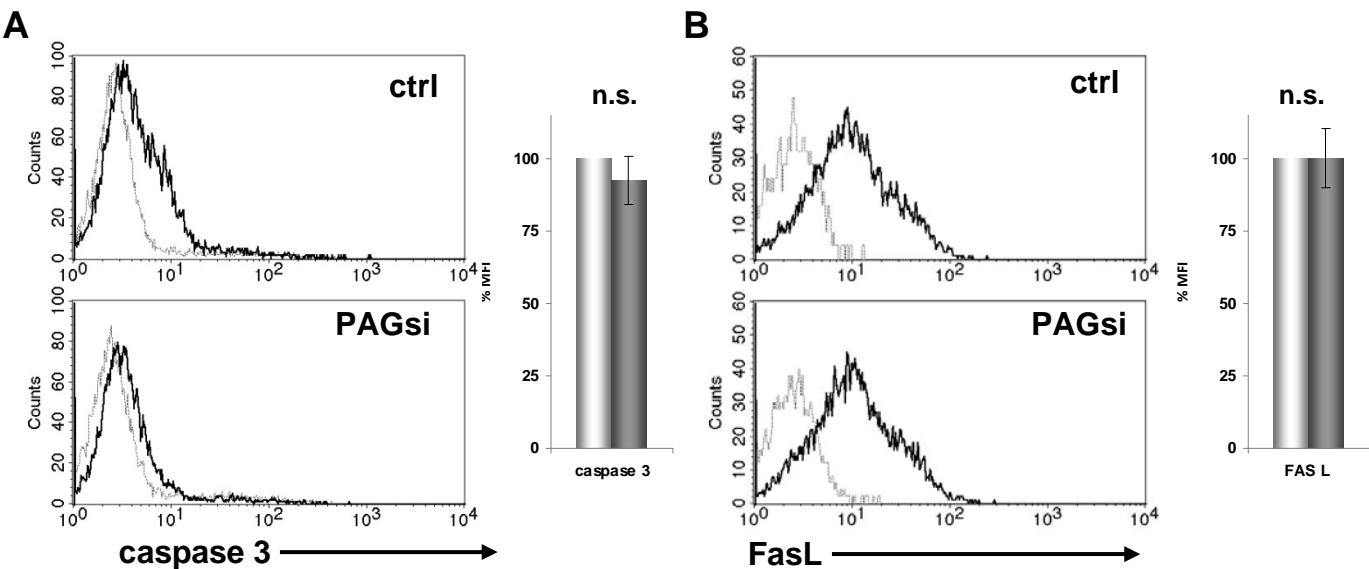

Supplement: Additional file 4: Figure S4 — Strong signaling does not induce apoptosis. Primary human T cells transfected either with Renilla (ctrl) or PAG (PAGsi) siRNA were stimulated on an anti-CD3+anti-CD28 coated plastic plate for three days. (A) The activation of caspase 3 was determined using FITC-conjugated DEVD-FMK. Profiles of unstimulated (grey line) versus stimulated (black line) cells are shown. White bars (ctrl), grey bars (PAGsi). (B) The upregulation of FasL was analyzed by flow cytometry. Profiles of unstimulated (grey line) versus stimulated (black line) cells are shown. Data are representative of three independent experiments. [file 1478-811X-11-28-S4.pdf]

Figure S6

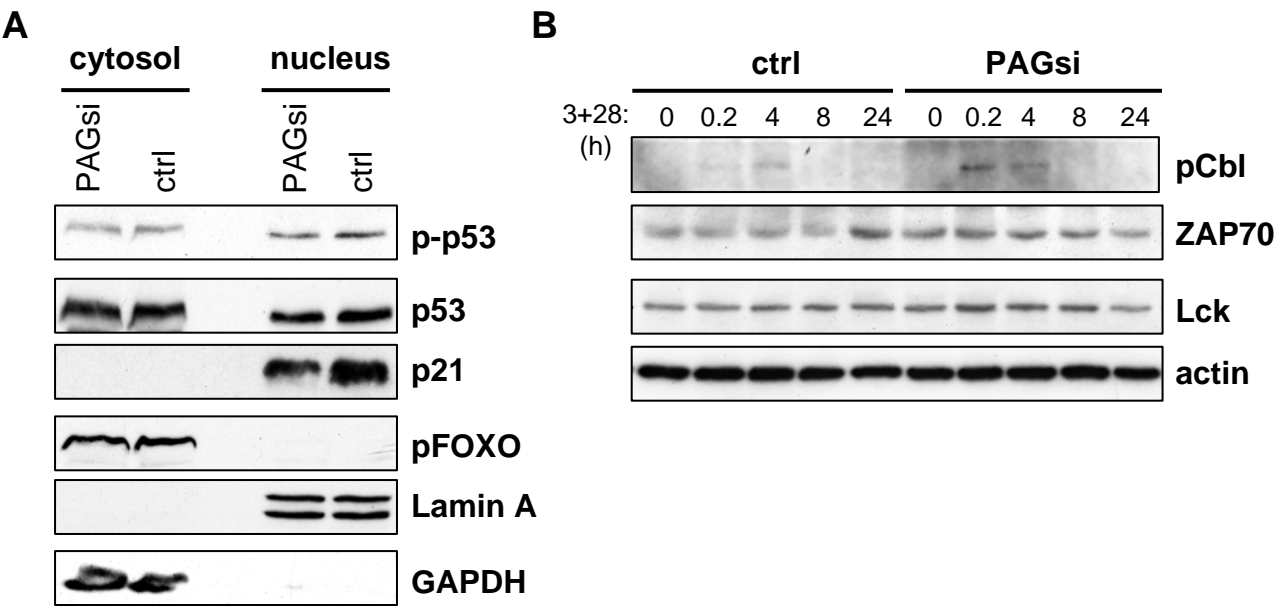

Supplement: Additional file 5: Figure S6 — T-cell unresponsiveness is not due to oncogene-induced senescence or enhanced Cbl activity. (A) Enhanced SFK activity does not result in oncogene-induced senescence. Primary human T cells transfected with Renilla (ctrl) or PAG (PAGsi) siRNAs were stimulated for 72 hours on an anti-CD3+anti-CD28 coated plastic plate. Isolation of cytoplasmic and nuclear fractions was performed as previously described (10). Briefly, cells were resuspended in an hypotonic buffer and incubated with 10% NP-40. After centrifugation at 2000 rpm, 5 min, 4oC, the cytoplasmic fraction was obtained. Pellets were washed and lysed in a stringent lysis buffer for 1 hour at 4oC with agitation. Samples were then centrifuged at 13000 rpm, 10 min, 4oC and the supernatant was taken as the nuclear fraction. Both fractions were loaded on a 12% acrylamide gel and immunoblotted with phospho-p53, total p53, total p21 [all from Exbio] and pFOXO1 antibodies [Cell Signaling]. Lamin A [BioLegend] and GAPDH [Abcam] antibodies were used as markers to detect nuclear and cytoplasmic fraction respectively. Data are representative of three independent experiments. (B) PAG suppression enhances phospho-Cbl, but does not affect Lck or ZAP-70 expression. Primary human T cells transfected with Renilla (ctrl) or PAG (PAGsi) siRNAs were stimulated with anti-CD3+anti-CD28 for up to 24 hours, lysed and immunoblotted for phosphorylation of Cbl (pY731) [Cell Signaling] and total expression of ZAP-70 [BD] and Lck [Biosource]. Actin staining is shown as a loading control. Data are representative of two independent experiments. [file 1478-811X-11-28-S5.pdf]

**Figure S5**

**Experiment 1**

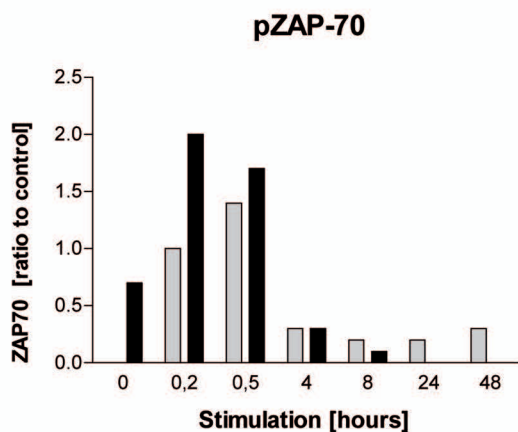

**Experiment 2**

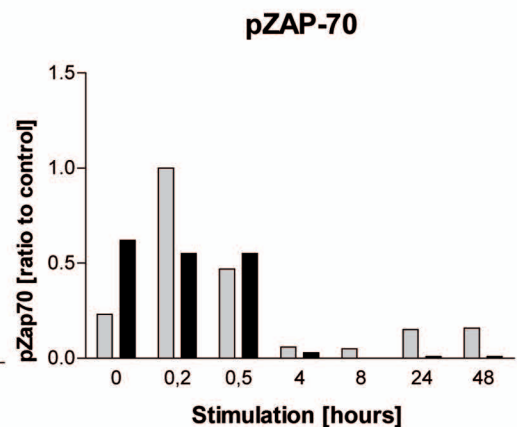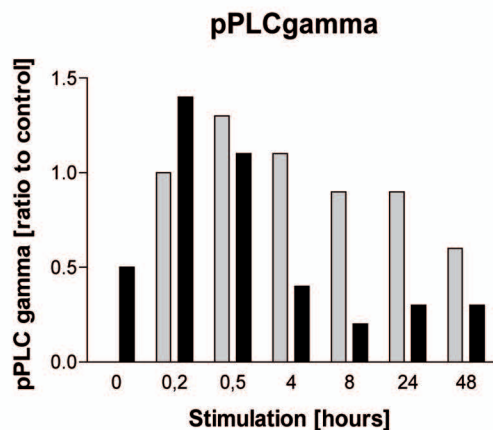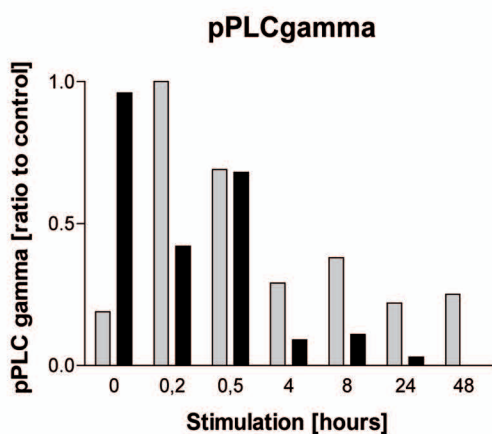

Supplement: Additional file 6: Figure S5 — Quantification of Figure 3. The relative signal intensities of p-ZAP-70 and p-PLCγ in Figure 3C were normalized to the loading control. The peak value of the control sample was set to 1.0. Data from two independent experiments are shown, in both cases the control samples show a sustained kinetic, whereas the signal in the PAG siRNA samples peaks earlier and terminates. [file 1478-811x-11-28-S6.pdf]

**Figure S7**

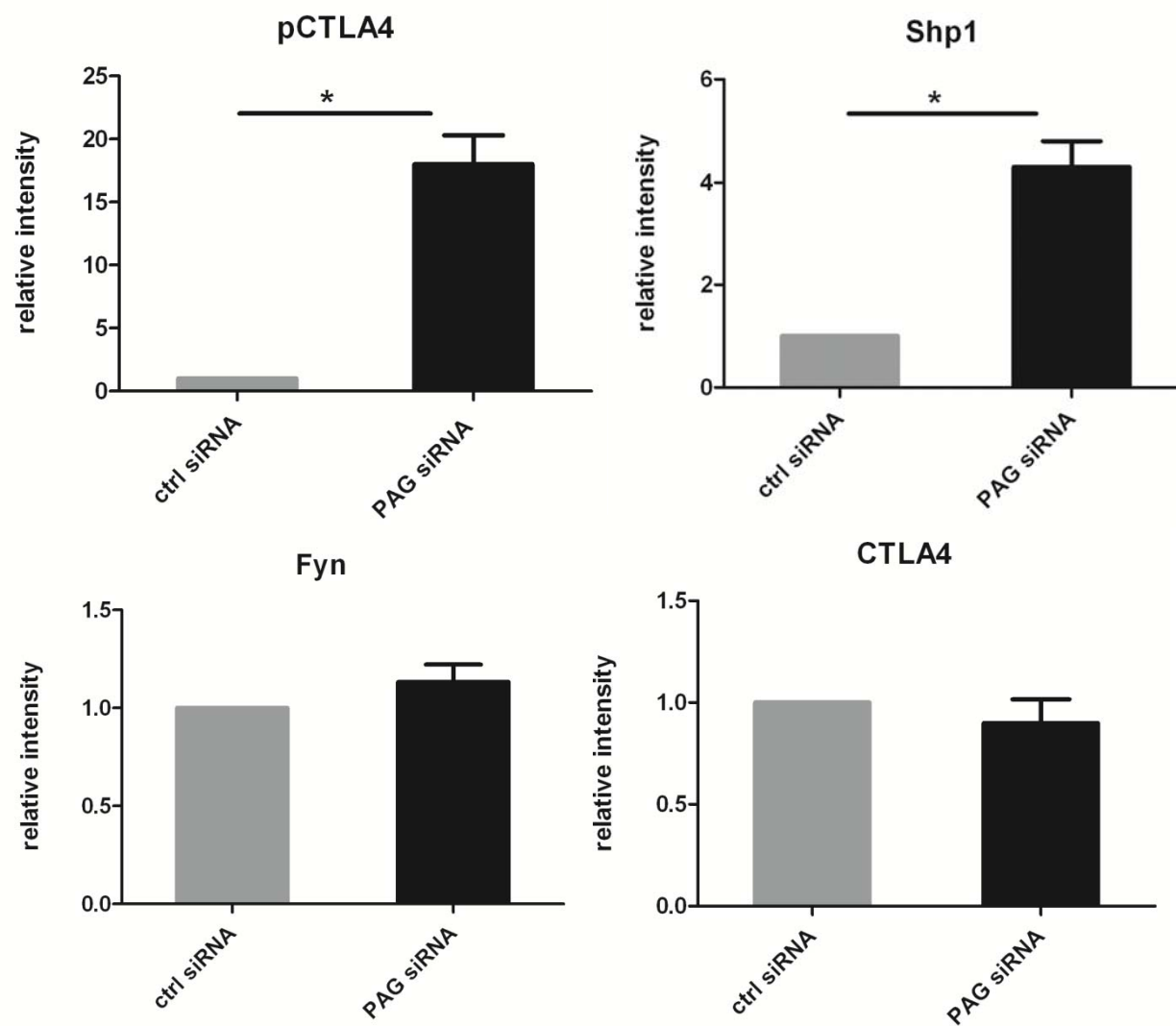

Supplement: Additional file 7: Figure S7 — Quantification of Figure 4. The blots from the CTLA-4 immunoprecipitates were analyzed. The control values set to 1.0 and a One-Sample t test analysis was performed (shown is the mean ± SEM; *, P<0.05, n = 4). As shown in Figure 4, a significant increase in the phosphorylation of CTLA-4 and recruitment of Shp1 is observed, while the amount of Fyn and CTLA-4 remain unchanged. [file 1478-811X-11-28-S7.pdf]

Figure S8

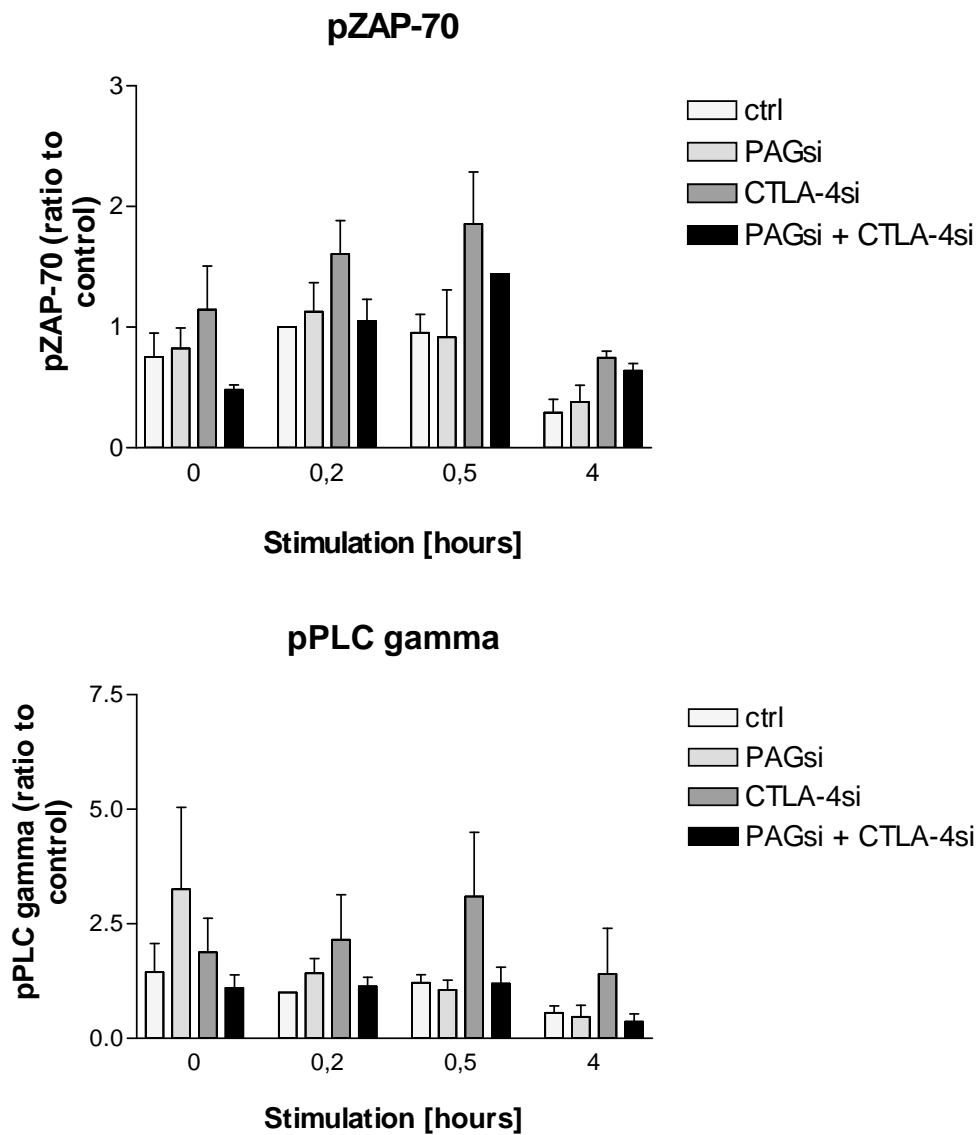

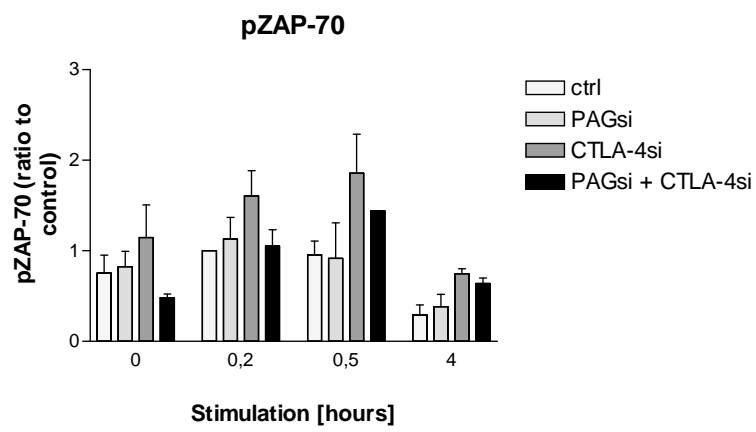

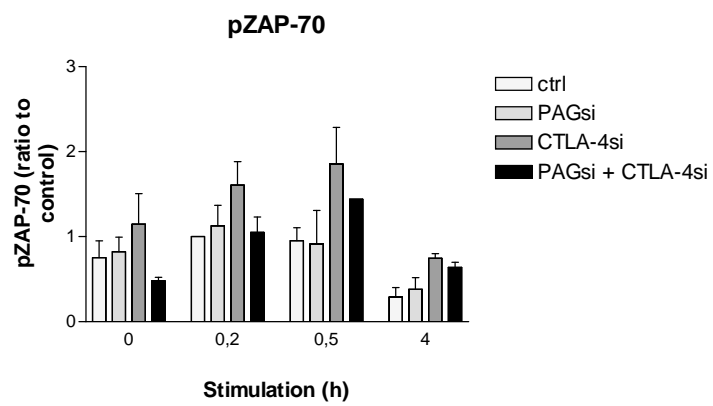

Supplement: Additional file 8: Figure S8 — Quantification of Figure 5. The relative signal intensities of p-ZAP-70 and p-PLCγ in Figure 5C were normalized with respect to the loading controls and the peak value set to 1.0 (the mean ± SEM is shown). Data are representative of at least three independent experiments. [file 1478-811X-11-28-S8.pdf]

Figure S9

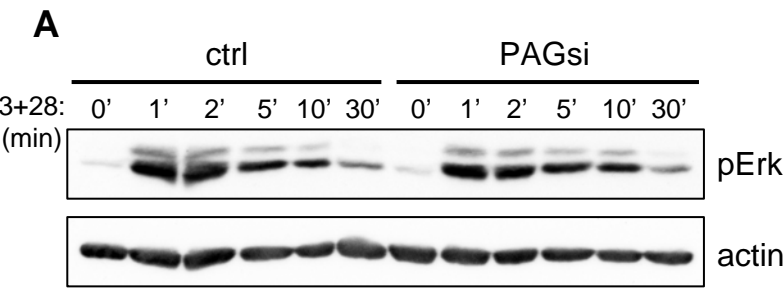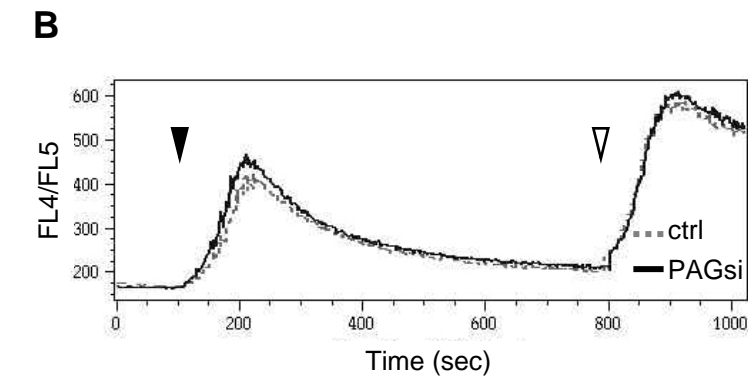

Supplement: Additional file 9: Figure S9 — PAG suppression does not affect TCR-induced ERK activation or calcium flux. (A) Jurkat T cells transfected either with control (ctrl) or PAGshRNA (PAGsi) constructs were stimulated with anti-CD3+anti-CD28 for the indicated time, lysed and immunoblotted for pERK [Cell Signaling]. Actin staining is shown for equal loading. Data are representative of three independent experiments. (B) Intracellular calcium flux was measured in primary human T cells transfected either with Renilla (ctrl; grey dashed line) or PAG siRNA (PAGsi, black solid line). Transfected T cells were washed with RPMI 1640 without phenol red and loaded with 5 μg/ml Indo-1 [Invitrogen] for 45 min at 37oC. The cells were washed briefly and incubated for an additional 30 min before measuring the FL4 (510/20 nm) versus FL5 (400/40 nm) ratio on an LSR1 flow cytometer [BD]. The cells were stimulated first with 50 μg CD3 (MEM-92) antibody supernatant and then with ionomycin (1 μg/ml). The addition of CD3 antibody and ionomycin is indicated by the filled and empty triangle, respectively. One representative experiment of three is shown. [file 1478-811X-11-28-S9.pdf]
